# Prevalence of Complementary Feeding Indicators and Associated Factors Among 6- to 23-Month Breastfed Infants and Young Children in Poor Rural Areas of China

**DOI:** 10.3389/fpubh.2021.691894

**Published:** 2021-10-01

**Authors:** Jing Liu, Junsheng Huo, Jing Sun, Jian Huang, Weiyi Gong, Ou Wang

**Affiliations:** Chinese Center for Disease Control and Prevention, National Institute for Nutrition and Health, Beijing, China

**Keywords:** complementary feeding, poor rural areas, minimum acceptable diet, breastfeeding, infants and young children

## Abstract

This study aimed to estimate the status of complementary feeding (CF) and its associated factors among 6–23-month breastfed infants and young children (IYC). We used secondary data from the China Nutrition Improvement Project on Children in Poor Areas in 2018. The status of CF was provided by parents of IYC through 24-h dietary recall. The study included 13,972 6–23-month-old breastfed IYC comprising 24.7% 6–8-month, 28.5% 9–11-month, 31.4% 12–17-month, and 18–23-month IYC. The highest percentage of IYC introduced to cereal foods was 84.8%. Nearly, 83.6% of 6–8-month infants were introduced to solid or semi-solid food. The prevalence of meeting requirements of non-dairy animal source food and minimum acceptable diet (MAD) was 75.3 and 35.1% of 6–23-month IYC, respectively, and was significantly higher in older than younger IYC (*p* < 0.001). Age of IYC, education level of parents, paternal employment, and nutrition knowledge of parents were positively associated factors for the prevalence of meeting requirements of MAD, and diarrhea at 2 weeks and maternal employment were negatively associated with MAD. Totally, the prevalence of meeting the requirements of MAD was relatively lower in breastfed IYC. The government should scale up appropriate CF with consideration of food availability.

## Introduction

Timely and adequate complementary food in IYC should be introduced between the ages of around 6 months and 2 years old and can affect nutritional and health status in later life (1, 2). The period from 6 months to 2 years is a crucial period in the life of a child in which appropriate complementary food can reduce infant morbidity and mortality and improve nutritional status, growth, and cognitive development (3, 4). Due to the small stomach capacity of infants, and because their nutrient needs reach a lifetime peak, they are vulnerable to faltering growth (2). Inappropriate complementary feeding (CF) during this critical window is a significant cause of malnutrition, which increases the risk of stunting, wasting, underweight, or overweight (5, 6). To improve the status of infant feeding, WHO and UNICEF have produced strategies and guidelines (2, 7, 8). Among these, strategies recommend that children are exclusively breastfed for 6 months after birth and provided safe and appropriate complementary foods with continued breastfeeding up to 2 years or beyond (7). To promote the infants and children complementary feeding (IYCF) recommendations of WHO, during the past decade, the Ministry of Health of China has developed programs to improve nutrition practices (9). However, infant feeding status remains unsatisfactory in China. In 2008, 27.6% of IYC were exclusively breastfed up to 6 months, 43.3% of 6–9-month children were introduced to solid or semi-solid foods, and 37.0% of 12–15-month children continued to be breastfed in China (10). From 2005 to 2015, the age at which a variety of complementary food was introduced was postponed to around 6 months. Simultaneously, the time of introduction in suburban IYC was close to that of urban IYC, with the exception of rice and fruit, by 2015 (11). Current indicators used to assess CF were first introduced by WHO, UNICEF, and other related institutes in 2008, including minimum dietary diversity (MDD), minimum meal frequency (MMF), and MAD (12). In 2013, the crude rates of MDD, MMF, and MAD were 52.5, 69.8, and 27.4%, respectively, for 6–23-month IYC in China (13). The status of CF was different in rural and urban areas, and the rates were the lowest in poor rural areas (13). As a result of rapid economic development, the status of CF practice might change. However, no research has focused on the CF status of breastfed IYC in poor rural areas.

In poor rural areas of China, few timely data exist to estimate the status of CF among breastfed IYC. Thus, our study focused on the breastfed IYC in poor rural areas. The first objective is to provide the general status of CF. The secondary objective is to estimate the associated factors of MAD.

## Materials and Methods

### Study Design and Population

The China Nutrition Improvement Project on Children in Poor Areas (CNIPCPA) was a national, yearly, cross-sectional survey of 6–23-month health IYC in poor rural areas that covered 148 counties in 19 provinces. In the program, samples were selected by complex sampling survey in various layers with a stratified sampling of provinces, multistage sampling, probability proportional to size (PPS) sampling, and systematic random sampling. At first, we chose provinces that had poor rural areas. Second, all the counties in each province were ranked from the lowest to the highest by gross domestic product (GDP) per capita. A certain number of counties were selected by the PPS sampling method, which was the sampling probability that was proportional to the size and was consistent with the number of live births in the county in 2012. In this step, we chose five to seven counties. Third, we ranked all the townships in each county according to their net income per capita. We selected five towns per county by the PPS sampling method. Fourth, we ranked all the villages in each town according to their net income per capita. We selected three to five villages per town by the PPS sampling method. At last, all IYC aged 6–23 months in the village were ordered according to their birth dates (from the youngest to oldest), and 12–20 IYC were selected by the systematic random sampling method. If the individual refused to participate or was unavailable, a replacement infant was chosen from the same village. Then, the IYC were undertaken a physical examination and their parents responded to the questionnaires face-to-face. Our study comprised a secondary data analysis, which focused on 6–23-month breastfed IYC. We included the IYC who were breastfeeding during the investigation. In total, there were 43,374 6–23-month IYC in our program in 2018. About 17,037 (39.3%) breastfed IYC were included in our study. The participants who had missing data (i.e., missing identification number, gender, age, and CF items) were excluded from the study. At last, there were 13,972 breastfed IYC in the analysis.

The Ethical Committee of the National Institute for Nutrition and Health of the Chinese Center for Disease Control and Prevention gave the approval to undertake this study. The Ethical Reference Number is 2014-001. All of the caretakers of the participants gave their informed written consent.

### Data Collection

We used questionnaires to collect data about the essential characteristics of the IYC, including sex and age. We also gathered information about their parents, including the level of education, ethnicity, and occupation. Furthermore, experts of CNIPCPA adapted a standard questionnaire based on WHO/UNICEF indicators to assess CF practice and measurement (8). The status of complementary food was collected through a 24-h dietary recall by the parents of IYC. We collected the frequency of eight groups of complementary food, namely, breastmilk; grains, roots, and tubers; legumes and nuts; dairy products (milk, infant formula, yogurt, and cheese); flesh foods (meat, fish, poultry, and liver/organ meats); eggs; vitamin A-rich fruits and vegetables; and other fruits and vegetables.

### Statistical Analysis

We carried out statistical analysis using IBM SPSS Statistics for Windows, version 26 (IBM Corp., Armonk, New York, USA). Continuous variables are presented as means and SD. Frequency and percentages are shown for binary or categorical variables, and chi-square tests were used to compare categorical variables. Multivariable logistic regression analysis was then conducted to assess associated factors of meeting requirements of MAD by estimating the odds ratios (OR) and corresponding 95% CIs. The logistic regression model included all the potential associated factors identified based on the chi-square test. The Hosmer Leme show of the goodness of fit test was 0.735, which indicated that the model fitted well. The R-square of the multivariable logistic analysis model was 0.123. All statistical tests were two-sided, and a significance level of <0.05 was specified.

The CF status of breastfed IYC was stratified and described by age and sex group. There are five indicators in the guide of UNICEF. MDD relates to 6–23-month IYC who receive foods from four or more food groups. MMF is defined as children who received solid, semisolid, or soft foods a minimum number of times the previous day (two times for breastfed 6–8 months and three times for 9–23-month breastfed children). MAD is a composite indicator based on 6–23-month IYC who had at least the MDD and the MMF during the previous day (8). Introduction of solid, semi-solid, or soft foods (ISSF) represents the proportion of IYC 6–8 months of age who receive solid, semi-solid, or soft foods. Non-dairy animal source food (NDASF) represents the percentage of 6–23-month IYC who consumed eggs and/or flesh foods during the previous day (2). In our study, breastfeeding was equal to “any breastfeeding,” that is, the IYC received breastmilk with or without other drinks, including formula, or other infant food (14). Fever and diarrhea were defined as self-reported by parents of IYC.

In the analysis, IYC were categorized into the following age groups: 6–8, 9–11, 12–17, and 18–23 months. We divided gestational age into two groups: more than 37 weeks and <37 weeks. Education of parents was categorized into two groups: graduated from middle school or above (middle/above) and primary school or below (primary/below). The ethnicity of the parents was divided into Han Chinese and other ethnic minorities. Occupation of parents was divided into two groups based on employment status. The nutrition knowledge score of caregivers was divided into two groups: ≥3 points and < 3 points.

## Results

### Characteristics of Infants, Parents, and Primary Caregivers

Table 1 shows the characteristics of breastfed IYC and their parents. There were 13,972 IYC aged 6–23 months in our study. There were 7,385 (52.9%) boys and 6,587 (47.1%) girls. Table 1 also shows the prevalence of diarrhea and fever within 2 weeks. More than three-fifth of the mothers were housewives, and the majority of the fathers were employed. The percentage of fathers who graduated from middle school or above was slightly more than that of the mothers. About 73% of parents were of Han ethnicity. Details of parents and IYC are shown in Table 1.

**Table 1:** Characteristics of infants and young children and their parents (*n* = 13,972).

**Characteristic**		**Sample *n* (%)**
**Infants**		
Age group		
	6–8 months	3,450 (24.7)
	9–11 months	3,988 (28.5)
	12–17months	4,388 (31.4)
	18–23months	2,146 (15.4)
Sex, *n* (%)		
	Boy	7,385 (52.9)
	Girl	6,587 (47.1)
Premature		472 (3.4)
Fever in 2 weeks		1,473 (10.5)
Diarrhea in 2 weeks		1,423 (10.2)
**Mothers**		
Nationality		
	Han	10,314 (73.8)
	Minorities	3,658 (26.2)
Education		
	Primary/below^a^	2,044 (14.6)
	Middle/above^b^	11,928 (85.4)
Employment		
	No	8,580 (61.4)
	Yes	5,392 (38.6)
**Fathers**		
Nationality		
	Han	10,301 (73.7)
	Minorities	3,671 (26.3)
Education		
	Primary/below^a^	1,635 (11.7)
	Middle/above^b^	12,337 (88.3)
Employment		
	No	2,682 (19.2)
	Yes	1,1290 (80.8)

a *Primary/below: graduated from primary school or below*.

b *Middle/above: graduated from middle school or above*.

### Consumption of Complementary Foods During a Single 24-h Dietary Recall

Table 2 shows the percentage and CI of seven food groups received by IYC during the previous day based on dietary recall during a single period of 24 h. In different age groups, CF was significantly different in various foods. As age increased, the percentage of introduction increased considerably. Grains, roots, and tubers were the most commonly consumed food for IYC, and the overall prevalence of introduction was 93.6%. The percentage of the introduction of dairy products, such as formula milk powder, yogurt, cheese, and milk powder, was 47.7%. Approximately, three-fifth of IYC consumed dark-colored vegetables and fruits, and about the same percentage consumed other vegetables and fruits. Only 29.0% of 6–8-month breastfed IYC were introduced to flesh foods, and the overall rate of flesh food introduction was 49.4%. The prevalence of egg consumption was 60.6%, which was higher than flesh consumption. About one-third of IYC consumed legume and nut products. As a supplementary, we also investigated the prevalence of sugar-sweetened beverage. A share of 16.7% of IYC consumed sugary beverages, and this prevalence increased with age (the prevalence was 7.3, 11.0, 21.2, and 33.0% in the four age groups, respectively).

**Table 2:** Percentage of complementary feeding for breastfed infants and young children during a single 24-h dietary recall period^a^.

	**Age group of IYC, % (CI)**				
**Food groups**	**6–8 months**	**9–11 months**	**12–17 months**	**18–23 months**	**Total, % (CI)**
**Sample number**	3450	3988	4388	2146	13972
Grains, roots, and tubers	89.6 (88.5, 90.6)	93.8 (93.0, 94.5)	95.0 (94.3, 95.6)	96.5 (95.7, 97.2)	93.6 (93.2, 94.0)
Dairy products^b^	34.7 (33.1, 36.3)	40.4 (38.9, 40.4)	54.0 (52.5, 55.5)	69.3 (67.3, 71.2)	47.7 (46.9, 48.5)
Flesh foods^c^	29.0 (27.5, 30.5)	44.5 (42.9, 46.0)	59.1 (57.7, 60.6)	71.7 (69.8, 73.6)	49.4 (48.6, 50.3)
Eggs	46.0 (44.4, 47.7)	58.2 (56.7, 59.8)	67.4 (66.0, 68.8)	74.6 (72.7, 76.4)	60.6 (59.8, 61.4)
Vitamin A-rich fruits and vegetables^d^	44.0 (42.4, 45.7)	57.4 (55.9, 58.9)	67.1 (65.7, 68.5)	76.5 (74.7, 78.3)	60.1 (59.3, 60.9)
Other fruits and vegetables^e^	43.3 (41.7, 45.0)	56.6 (55.1, 58.2)	64.5 (63.0, 65.9)	69.2 (67.2, 71.1)	57.7 (56.9, 58.5)
Legumes and nuts^f^	16.6 (15.4, 17.8)	29.1 (27.7, 30.6)	45.1 (43.6, 46.6)	58.3 (56.2, 60.4)	35.5 (34.7, 36.3)

a *Values are frequency and percentage of IYC consuming the food during a single 24-h recall period*.

b *Includes milk, infant formula, yogurt, and cheese*.

c *Includes meat, fish, poultry, and organ meats*.

d *Includes dark-green, orange, red vegetables, and fruits, and red or orange tuber crops*.

e *Includes other vegetables and fruits (e.g., eggplant, cauliflower, mooli, pear, and apple)*.

f *Includes bean and its products, walnut, cashew, almond, melon seeds, peanut, and its products*.

### The Prevalence of Complementary Feeding Indicators in Infants and Young Children

Table 3 shows the association between the introduction of solid/semi-solid food (ISSF), MDD, MMF, MAD, and NDASF, and age group and sex. About 83.6% of 6–8-month IYC were ISSF. Prevalence of NDASF, MDD, MMF, and MAD was 75.3, 58.5, 51.6, and 35.1%, respectively, for 6–23-month breastfed IYC. Prevalence of MAD was only 16.4% in 6–8-month breastfed IYC. About three-fifth of 6–8-month IYC were fed with egg and/or flesh foods. All of the CF indicators were significantly higher in older IYC than younger groups (*p* < 0.001). There was no difference between boys and girls in these indicators.

**Table 3:** Complementary feeding indicators of breastfed infants aged 6–23 months.

**Characteristic**		**ISSF** ^**a**^	**MDD** ^**b**^	**MMF** ^**c**^	**MAD** ^**d**^	**NDASF** ^**e**^
Age group						
	6–8 month	3,713 (83.6)	1,201 (34.8)	1,224 (35.5)	565 (16.4)	2,048 (59.4)
	9–11 month	NA	2,171 (54.4)	1,992 (49.9)	1,194 (29.9)	2,959 (74.2)
	12–17 month	NA	3,079 (70.2)	2,618 (59.7)	1,977 (45.1)	3,651 (83.2)
	18–23 month	NA	1,729 (80.6)	1,380 (64.3)	1,165 (54.3)	1,868 (87.0)
	Total	NA	8,180 (58.5)	7,214 (51.6)	4,901 (35.1)	10,526 (75.3)
	Chi-square	NA	1501.582	616.347	1115.558	781.119
	*p*-value	<0.001	<0.001	<0.001	<0.001	<0.001
Sex						
	Boy	1,948 (83.2)	4,780 (58.8)	3,797 (51.2)	2,566 (34.7)	5,563 (75.3)
	Girl	1,765 (84.0)	4,297 (58.7)	3,434 (51.9)	2,335 (35.4)	4,963 (75.3)
	Chi-square	0.053	0.084	0.609		0.001
	*p*-value	0.879	0.791	0.729	0.385	0.981

a *ISSF: Introduction of solid, semi-solid, or soft foods*.

b *MDD: Minimum dietary diversity*.

c *MMF: Minimum meal frequency*.

d *MAD: Minimum acceptable diet*.

e *NDASF: Nondairy animal source food*.

### Comparison of Characteristics of Infants and Young Children and Parents Between the Qualified and Non-qualified Minimum Acceptable Diet Groups

Table 4 shows the chi-square univariate analysis results of CF factors in the MAD group compared with the reference group. The prevalence of MAD of 16.4% in the 6–8-month age group was far lower than that of the 12–17-month group (45.1%) and 18–23-month age group (54.3%) (*P* < 0.001). Prevalence of MAD was higher in IYC whose parents had middle/above educational level than those with primary/below (*P* < 0.001). Among the paternal occupation, the prevalence of MAD in IYC with paternal employment of 35.9% was higher than the 31.8% in the reference group (*P* < 0.001). In the MAD group, the prevalence in those that were diarrhea within 2 weeks was lower than that in the group that was not sick. Those with more nutritional knowledge scores showed a higher prevalence of MAD than those with a lower score (*P* < 0.001).

**Table 4:** Results of univariate analysis between qualified and nonqualified minimum acceptable diet groups.

	**Non-MAD**	**MAD**	**Chi/F**	***P*-value**
**Age group**			1115.56	<0.001
6–8 months	2,885 (83.6)	565 (16.4)		
9–11 months	2,794 (70.1)	1,194 (29.9)		
12–17 months	2,411 (54.9)	1,977 (45.1)		
18–23 months	981 (45.7)	1,165 (54.3)		
**Sex**			0.75	0.39
Boy	4,819 (65.3)	2,556 (34.7)		
Girl	4,252 (64.6)	2,335 (35.4)		
**Premature** ^**a**^			5.63	0.06
No	8,745 (64.8)	4,747 (35.2)		
Yes	318 (67.4)	154 (32.6)		
**Fever in 2 weeks** ^**b**^			3.22	0.20
No	8,086 (64.7)	4,404 (35.3)		
Yes	980 (66.5)	493 (33.5)		
**Diarrhea in 2 weeks**			19.92	<0.001
No	8,071 (64.3)	4,478 (35.7)		
Yes	1,000 (70.3)	423 (29.7)		
**Maternal education**			75.30	<0.001
Primary/below	1,500 (73.4)	544 (26.6)		
Middle/above	7,571 (63.5)	4,357 (36.5)		
**Maternal employment**			0.12	0.73
No	5,561 (64.8)	3,019 (35.2)		
Yes	3,510 (65.1)	1,882 (34.9)		
**Paternal education**			56.68	<0.001
Primary/below	1,198 (73.3)	437 (26.7)		
Middle/above	7,873 (63.8)	4,464 (36.2)		
**Paternal employment**				<0.001
No	1,830 (68.2)	852 (31.8)		
Yes	7,241 (64.1)	4,049 (35.9)		
**Nutrition knowledge score**			289.01	<0.001
<3	3,108 (75.5)	1,006 (24.5)		
≥3	5,963 (60.5)	3,895 (39.5)		

a *Premature: There were 8 IYC have no information about this*.

b *Fever in 2 weeks: There were 5 IYC have no information about this*.

### Multivariable Logistic Analysis of Minimum Acceptable Diet Prevalence in 6–23-Month Breastfed Infants and Young Children

All independent variables were analyzed by chi-square test, and significantly independent variables were included in multivariable logistic regression analysis. Occupation of mothers was an important factor in CF that we included in this analysis. Table 5 details the results of the multivariable logistic analysis of factors associated with the prevalence of MAD in breastfed IYC. IYC in the 9–11-month age group (OR = 2.22, 95% CI 1.98, 2.48), 12–17-month age group (OR = 4.29, 95% CI 3.85, 4.78), and 18–23-month age group (OR = 6.23, 95% CI 5.50, 7.06) were associated with having higher prevalence of meeting MAD requirement. IYC who experienced diarrhea in 2 weeks (OR = 0.86, 95% CI 0.78, 0.95) were associated with having a lower prevalence of MAD. Mothers and fathers graduating from middle school or above were a protective factor of MAD compared with parents who graduated from primary school or below. Employment of fathers (OR = 1.27, 95% CI 1.15, 1.40) showed a significant association with a higher prevalence of MAD. However, mothers who worked showed a lower prevalence of MAD. Caregivers with a nutrition knowledge score higher than three were a protective factor for meeting the requirements of MAD.

**Table 5:** Multivariable logistic analysis of the minimum acceptable diet prevalence.

**Independent variables**	**β**	**SE**	**OR (95% CI)**	***p*-value**
**Age group**				<0.001
6–8 months	1			
9–11 months	0.795	0.058	2.22 (1.98, 2.48)	<0.001
12–17 months	1.456	0.055	4.29 (3.85, 4.78)	<0.001
18–23 months	1.830	0.064	6.23 (5.50, 7.06)	<0.001
**Diarrhea in 2 weeks**				<0.001
No	1			
Yes	−0.152	0.050	0.86 (0.78, 0.95)	0.002
**Maternal education**				<0.001
Primary/below	1			
Middle/above	0.385	0.068	1.47 (1.29, 1.68)	<0.001
**Maternal employment**				<0.001
No	1			
Yes	−0.097	0.041	0.91 (0.84, 0.98)	0.018
**Paternal education**				<0.001
Primary/below	1			
Middle/above	0.196	0.075	1.22 (1.05, 1.41)	<0.001
**Paternal employment**				<0.001
No	1			
Yes	0.238	0.052	1.27 (1.15, 1.4)	0.018
**Nutrition knowledge score**				
<3	1			
≥3	0.119	0.038	1.13 (1.05, 1.21)	0.002

## Discussion

Our study used UNICEF CF indicators to explore the feeding status of breastfed IYC in poor rural areas of China. The primary outcome was to investigate the prevalence of CF, which includes NDASF, MDD, MMF, and MAD, which were still low, but they improved with age. The secondary outcome was estimated associated factors underlying meeting requirement of MAD among 6–23-month breastfed children in rural areas of China in 2018. About 83.6% of IYC had been ISSF. CF indicators were significantly different in different age groups. Age of the breastfed IYC, parents graduated from middle school or above, fathers were employed, and nutrition knowledge score more than three were protective factors for the prevalence of meeting MAD requirements. IYC who experienced diarrhea in 2 weeks and whose mothers were employed were associated with the lower prevalence of MAD.

Good CF provides adequate nutrition to IYC, influences their food preferences and eating behavior, and helps shape healthy dietary habits (15). In our study, we showed the percentage intakes of eight types of foods. Foods made from cereals are the main food of IYC. Based on the WHO recommendation, IYC should be introduced to a daily intake of varied and adequate quantities of meat, poultry, and fish or eggs, in addition to fruits and vegetables rich in vitamin A (1, 8). Unfortunately, previous studies have shown that inappropriate feeding practices for 6–23-month IYC are common in many rural areas of China (16). For instance, complementary foods generally comprise carbohydrates and lack protein and fat (17). In this study, 18.5% of 6–23-month IYC consumed sugary beverages, and this percentage increased in older age groups. Sipping sugary drinks can lead to a desire for food with a sweet taste and the rejection of foods with other tastes (18, 19). A prospective cohort study indicated that delayed introduction of sweet food intake at 1 year predicted better diet quality at 3 years (20).

It is crucial to promote good nutrition in the early stages of life, including dietary diversity, meal frequency, and acceptable diet, because malnutrition could cause irreversible damage. However, the results of our study showed that the prevalence of meeting requirements of MDD, MMF, and MAD could be improved in 6–23-month breastfed IYC in poor rural areas. The guide of UNICEF includes breastmilk as a type of food, and the MDD in breastfed IYC was higher than that of previous results (2). The status of CF was better than that in several Asian countries, such as India, Nepal, Bhutan, and Pakistan (21). Furthermore, it was significantly improved compared with results obtained in 2013, in which the rates of meeting requirements of MDD, MMF, and MAD in poor rural areas were only 40.5, 45.6, and 9.5%, respectively (13). In 6–8-month IYC, the percentage meeting ISSF requirements was 83.6%, and the age of introduction might affect the outcome of overweight and obesity in the later life (22). Results from IYC aged 0–24 months showed that the frequency of CF affected the body weight of IYC due to lack of adequate nutrition (23). In many countries, indicators of diet diversity and overall diet quality are positively associated with height for age (HAZ) (24). In many poor areas of China, inadequate MAD was positively associated with a higher stunting rate among children aged 3 years (25). Based on 11 demographic and health surveys, previous research studies found that dietary diversity was significantly associated with HAZ and stunting (26). Previous studies have also illustrated that a lack of dietary diversity will contribute to symptoms of malnutrition in IYC, and particularly stunting (26–28). To address the problem of malnutrition in 6–23-month IYC, the government and relevant departments should promote and enhance education related to CF in poor rural areas.

Our findings revealed that IYC of parents with higher education levels were more likely to meet MAD requirements than parents with primary school or below education. Parents with a lower education level may have a low salary and little knowledge of feeding practices. Due to the lack of sufficient food supplies and scientific feeding knowledge, parents might only introduce grain foods as complementary food and breast milk to IYC (25). Some caregivers believed that IYC could not digest meat and some do not know how to cook meat for IYC (29). To improve feeding patterns of IYC, caregivers should be provided feeding knowledge and helped to cook nutritious food for their children based on local products (30). The rate of MDD among IYC in the wealthiest households is two times lower than that in the most impoverished families at the global level (31). Parents with higher education level and paternal employment may face better economic conditions. It is crucial to raise the income of families to improve infant feeding practices. Due to better economic conditions, nutrition education and CF counseling for parents improved effectively the feeding pattern in rural areas (32). However, maternal employment was negatively associated with MAD. Working mothers may not have enough time to pay attention to their children due to work and home pressures. The Chinese government has committed to poverty alleviation in impoverished areas to improve the quality of life and the nutrition status of IYC.

Our research was well-designed and well-conducted. It covered a major aspect reflecting one of the causes of breastfed IYC malnutrition and finding the associated factors among adequate sample size. To impact social health, policymakers can promote implementation policy in a positive direction based on our results. We used the latest UNICEF CF indicators to assess the prevalence of qualified feeding among breastfed IYC in Chinese poor rural areas. The data of the study were timely and representative. Limitations of the study are that it is based on 24-h recall, the reliability of which is questionable at the 7-day history that should be taken preferably. Our results were relevant to breastfed IYC only. In addition, the age of introduction of complementary foods and food availability status were not included in our questionnaire.

## Conclusions

In conclusion, the primary outcome of our study is providing the prevalence of meeting NDASF, MMF, MDD, and MAD requirements, which is found to be increased with age among 6–23-month breastfed IYC in poor rural areas of China. The secondary outcome of the study is estimating the associated factors of MAD. Age of IYC, education level, paternal employment, and nutrition knowledge of parents were positively associated factors for the prevalence of meeting MAD requirements. Diarrhea at 2 weeks and maternal employment are negatively associated with MAD. The government should implement policy in conjunction with the local culture to scale up and appropriate CF, with consideration of food availability, among 6–23-month IYC in China.

## Data Availability Statement

The datasets presented in this article are not readily available because of the regulations of Data Management in Chinese Center for Disease Control and Prevention. Requests to access the datasets should be directed to National Institute for Nutrition and Health, Chinese Center for Disease Control and Prevention, which email address is chinanutrition@chinacdc.cn.

## Author Contributions

JL, WG, and OW contributed to the methodology. JHuo, JS, and JHua contributed to resources. JL contributed to the first draft of the manuscript and contributed to review and editing. All authors contributed to manuscript revision and approved the submitted version.

## Conflict of Interest

The authors declare that the research was conducted in the absence of any commercial or financial relationships that could be construed as a potential conflict of interest.

## Publisher's Note

All claims expressed in this article are solely those of the authors and do not necessarily represent those of their affiliated organizations, or those of the publisher, the editors and the reviewers. Any product that may be evaluated in this article, or claim that may be made by its manufacturer, is not guaranteed or endorsed by the publisher.
